# Promising Response of Paclitaxel in Metastatic Transitional Cell Carcinoma of Distal Ureter Complicated with Hydroureteronephrosis: A Case Report

**DOI:** 10.31729/jnma.8072

**Published:** 2023-03-31

**Authors:** Sajjad Ahmed Khan, Santosh Sah, Surya Bahadur Parajuli, Sulav Sapkota, Asmita Rayamajhi

**Affiliations:** 1 Birat Medical College and Teaching Hospital, Biratnagar, Morang, Nepal; 2 Department of Community Medicine, Birat Medical College and Teaching Hospital, Biratnagar, Morang, Nepal; 3 Department of Medical Oncology and Haematology, Birat Medical College and Teaching Hospital, Biratnagar, Morang, Nepal

**Keywords:** *carcinoma*, *case reports*, *metastasis*, *paclitaxel*

## Abstract

Ureteric carcinoma is the rarest of all urothelial malignancies, and little attention has been given to it. Palliation in these groups of patients is a dilemma in the clinics. Use of chemotherapeutic agents in ureteric carcinoma is a double edged sword, as these patients had already impaired renal function due to post-renal failure and nephrotoxic nature of most of the chemotherapeutic agents can further deteriorate the renal function, making the management approach, a relatively visionary task. Here, we present a case of a 77-year-old female with metastatic ureteric carcinoma locally complicated with hydroureteronephrosis, coming to us with gross haematuria, lower abdominal pain along with cough. Apart from age factor of the lady, presence of hydroureteronephrosis and pulmonary metastases was another challenge for us. Paclitaxel remains the mainstay of our treatment.

## INTRODUCTION

Transitional cell carcinoma (TCC) can occur anywhere in the urinary tract.^1^ Depending upon the site of primary tumor TCC has been divided into upper tract malignancies involving pyelocaliceal system and ureter, and lower tract malignancies affecting bladder and urethera.^2^ Upper tract malignancies are rare compared to urothelial cancer of the lower tract, comprising only 5-10% of all urothelial cancers.^3^ Amongst the upper tract TCC, ureteric carcinoma is the rarest of all accounting for 0.9-1.6% of all urothelial malignancies.^4^ The average survival rate in metastatic upper tract TCC is 17 months.^5^ Here, we present a case of 77-year old female with metastatic transitional cell carcinoma responsed with Paclitaxel.

## CASE REPORT

A 77-year-old-lady came up with chief complaints of lower abdominal pain for five months, dry cough for four months and intermittent gross hematuria lasting one to two days occurring almost every month since past four months. Hematuria was throughout the urine flow, with passage of fresh blood without clots.

Lower abdominal pain was dull aching, non-radiating with no aggravating or relieving factors. There was no history of burning micturition, increased frequency of urination, urgency, nocturia, weight loss, weakness, hemoptysis, waxing and waning of pain. There was no history of any chronic medical illnesses. Surgical history is significant for hysterectomy done for obstetric indication. No history of similar illnesses or any co-morbid conditions in the family.

On examination, there was presence of pallor, tachycardia and swelling of the lower extremities. Vitals were within the normal limits except for mild hypotension. On per abdominal examination, there was distension of the abdomen, kidney was palpable, dull note was heard over lower abdominal region. Respiratory examination revealed bilateral decreased chest expansion, while, cardiovascular, neurological and all other systemic examinations were within normal limit.

**Figure 1:**
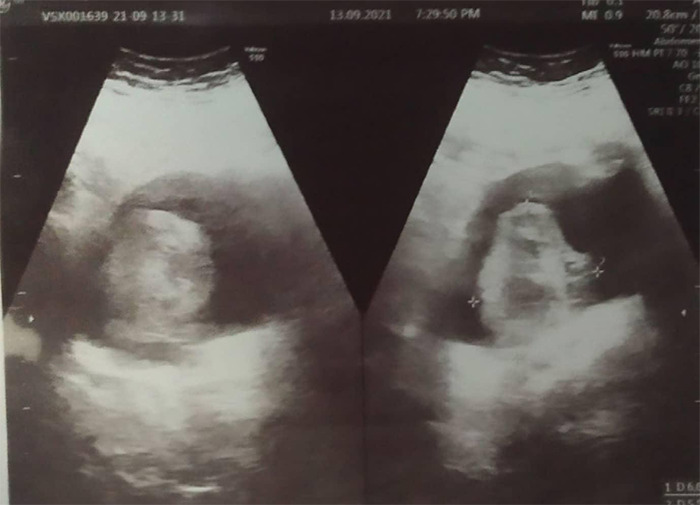
Gross hydroureteronephrosis in right kidney and right ureter is dilated up to the lower end with ill-defined large mass lesion and cystitis.

Suspecting it to be some urinary tract pathologies, ultrasonography (USG) of pelvis was done which showed gross hydroureteronephrosis in right kidney with marked cortical thinning measuring 4 mm in thickness. Right ureter was dilated up to the lower end with ill-defined large mass lesion measuring 66.9 x 55.9 mm at right vesicoueteric junction involving distal ureter and having intravesical component. Urinary bladder shows thickened wall measuring 4.1 mm with evidence of cystitis. USG abdomen revealed grade II fatty liver.

Computed tomography (CT) scan of abdomen showed bulky left adrenal gland, moderate to gross hydroreteronephrosis in right kidney with mild cortical thinning measuring 4 mm in thickness. Right ureter is dilated upto the lower end with an ill-defined hypodense mass lesion measuring 30 x 24 mm at right vesicoureteric junction. Bilateral kidney shows multiple simple cyst measuring 2-7 mm. Left kidney showed normal contrast excretion with contrast opacification of bilateral ureter and urinary bladder.

After this magnetic resonance urography (MRU) was done which showed circumferential segmental thickening of the wall of right distal ureter extending up to vesicoureteric junction with moderate to marked heterogeneous enhancement with moderate to marked right hydroureteronephrosis and regional lymphadenopathy was revealed, highly suspicious of transitional cell carcinoma. To arrive at a definitive diagnosis, biopsy was taken and sent for histopathological evaluation and findings were suggestive of high grade urothelial carcinoma. Further, on positron emission tomography (PET) scan, right common iliac lymph node involvement was detected.

Following the confirmation of the diagnosis, we decided to go ahead with injection paclitaxel 100 mg on monthly basis. We have planned for total of 18 cycles and patient is currently on the fourteenth cycle of the drug. Talking about the pre-chemotherapeutic agents, injection ranitidine 50 mg, injection ondansetron 8 mg and injection dexamethasone 8 mg with 100 ml normal saline were used to avoid any kind of gastro-intestinal intolerance and to counteract drug induced emesis. Injection paclitaxel 100 mg with 250 ml normal saline was infused over 2 hours. Also, as we planned for long-treatment with paclitaxel, hemorrhagic cystitis was the likely complication anticipated, for which we added injection mesna 400 mg to our protocol.

Till now, the patient is on the same medication protocol and doing well with the drugs without any apparent toxicity. She is able to give continuity to her daily routine activities with minimal difficulties and marked clinical as well as radiological improvement has been noted after the initiation of chemotherapy.

## DISCUSSION

Palliative care in metastatic ureteric carcinoma is a constantly evolving topic in medical oncology. As per the current guidelines, recommended palliative treatment in these group of patients is platinum-based combination chemotherapy, especially using cisplatin, however not all patients can go for it because of impaired renal function and cisplatin can further deteriorates the renal function because of its nephrotoxic nature, which may also significantly reduce the survival of the patients.^6^ Though, there are a handful of literatures on palliative surgical procedures in these group of patients, we refrained from any sort of operative procedures in this lady. Paclitaxel acts by supressing vascular endothelial growth factors (VEGF) and thus exerting anti-angiogenic effect, another proposed mechanism is it induces reactive oxygen species (ROS) generation and increases hydroperoxide production by enhancing the activity of nicotinamide adenine dinucleotide phosphate (NADPH) oxidase, which contributes to oxidative stress and demise of the malignant cells.^7^ Development of targeted therapy is still not very successful in case of urothelial malignancies, however, human epidermal growth factor receptor 2, p53, Hsp27, tubulin, cytotoxic T-lymphocyte antigen 4, VEGF, CD105, pololike kinase-1, phosphatidylinositide 3-kinases (PI3K), transforming growth factor β receptor/activin receptorlike kinase β, estrogen receptor, and the hepatocyte growth factor receptor (HGFR or MET) are novel targets in clinical trials.^8^ Talking about the use of paclitaxel in urothelial malignancies, a case of bladder cancer with hepatic and pelvic metastases reported from China, showed marked reduction in metastatic radiological features and clinical symptoms, when assessed after three cycles of paclitaxel based chemotherapy^9^

Inadequacy of knowledge regarding the molecular biology of these tumours is the major limiting factor for development of targeted therapies. Genomic profiling and molecular assessment of the tumor is neither available in everywhere, nor every patients can afford for it. In context of Nepal, the scenario is even worse, lack of trained oncologists, unavailability of proper diagnostic and treatment modalities, lack of public awareness about the disease processes, ignorance about the clinical features at early stage, presenting at a very advance stage and poor compliance to treatment and follow-up are few other inevitable factors making the treatment further challenging. The team managed this case with the diagnostic and treatment modalities which are feasible in our resource poor setting. She is about to complete 2 years with metastatic ureteric tumour. Till now we have not made any change in the treatment strategies, follow-up radiology findings have shown marked diminution of the metastatic and primary tumor foci. Signs and symptoms of the disease have been minimized significantly and she is able to carry out her daily routine activities.
